# Telemedicine in home-based care for COVID-19 patients

**DOI:** 10.1186/s12875-023-02199-y

**Published:** 2023-11-29

**Authors:** Si Van Nguyen, Huong Nguyen Viet Duong, Hieu Bao Nguyen, My Ai Thao Doan, Duc Thanh Nguyen, An Tuan Tran, Khoi Kim Hoang, Oanh Hoang Ly, Thanh Xuan Dang, Tung Ho Thanh Tran, Hung Quang Tran, Nam Ba Nguyen, Thuy Thi Thu Nguyen, Raghu Rai, An Le Pham

**Affiliations:** 1 Faculty of Medicine, University of Medicine and Pharmacy at Ho Chi Minh City, Ho Chi Minh City, Vietnam; 2 Jio Health Smart Clinic, Ho Chi Minh City, Vietnam

**Keywords:** COVID-19, Delta, Omicron, Telemedicine, Home-based care

## Abstract

**Background:**

The COVID-19 pandemic has made devastating impacts on public health and global economy. While most people experience mild symptoms, it is highly transmissible and deadly in at-risk populations. Telemedicine has the potential to prevent hospitalization and provide remote care.

**Methods:**

This retrospective study included 336 people with COVID-19, among which 141 (42%) and 195 (58%) were in Delta and Omicron dominant groups, respectively. Patients were confirmed to have COVID-19 by PCR or rapid test and were cared for via telemedicine. Severe cases were hospitalized for more intensive treatment.

**Results:**

The majority of individuals recovered at home (97.02%), while 2.98% required hospitalization. All hospital admissions were in Delta dominant group. No deaths were reported. Delta dominant group was more likely to develop loss of taste and smell, decreased appetite and need longer treatment time than those in Omicron dominant group.

**Conclusions:**

Telemedicine is a safe measure to provide at-home care for people with COVID-19 infections caused by both Delta and Omicron variants.

**Trial registration:**

This study was approved by the Institutional Review Board Committee of University of Medicine and Pharmacy at Ho Chi Minh City (IRB No: 22115–DHYD).

**Supplementary Information:**

The online version contains supplementary material available at 10.1186/s12875-023-02199-y.

## Background

The coronavirus pandemic is arguably the most serious global health crisis of the 21^st^ century. As of July 2022, more than 10.7 million people in Vietnam had been infected [1], with the recent fourth wave starting as the most disruptive yet. It developed in April 2021 and was marked by the emergence of the fast-spreading and highly contagious Delta variant. Across the country, Ho Chi Minh City swiftly became the epicenter of the COVID-19 pandemic, with medical institutions overloaded with infected cases and excess mortality seen in critically ill patients [2]. Seven months later, in November 2021, Omicron variant, which was less fatal and rendered the disease more manageable but highly contagious, took over [3]. Nevertheless, the pandemic remains a global health and economic crisis with long-term impacts on the community.

Telemedicine is the remote delivery of healthcare services via telecommunication platforms to serve patients and clients [4]. Its role during the pandemic has been manyfold, from triaging patients with severe illnesses who require early hospitalization to providing care for those with mild or asymptomatic COVID-19 which account for 80% of all cases and could be safely followed up at home [5, 6]. In this respect, it has been proven as an effective and satisfactory tool to prevent unnecessary hospital visits and the spread of the pandemic. This study was conducted to examine the utility and safety of telemedicine in caring for people with COVID-19 during both Delta and Omicron variants’ dominant periods.

## Methods

### Study settings

This retrospective study included patients diagnosed with COVID-19 by a rapid screening or polymerase chain reaction (PCR) test and received care via Jio Health app-based telemedicine from August 2021 to March 2022; pregnant people were excluded. During the study period, Delta variant was dominant among infected patients from August to December 2021 and was subsequently replaced by Omicron variant from January to March 2022, according to the country's COVID-19 situation report submitted to the World Health Organization (WHO) as of March 13, 2022 [7, 8].

### About Jio Health

Jio Health Polyclinic Company Limited is a private health clinic licensed by the Ho Chi Minh City Department of Health (License No: 0309145924) and a training unit of the Family Medicine Department of the University of Medicine and Pharmacy (UMP) in HCMC. Our telemedicine service is compliant with the regulations of Vietnam's Ministry of Health and is encrypted to protect clients' medical information. The platform can be downloaded online to smart mobile devices using Android or iOS operating systems from Google Play or App Store, respectively.

### Statistical analysis

All computations were done using IBM SPSS Statistics 20. The Shapiro–Wilk and skewness-kurtosis tests were used to check for normality. Normally distributed continuous variables are presented as mean and standard deviations, while non-normally distributed continuous variables are shown as median and interquartile range. Sample sizes and percentages are used to represent categorical variables. Statistic tests used to compare the characteristics of patients among Delta and Omicron dominant groups were the Chi-square test for categorical variables and the t-test of Mann–Whitney U test for continuous variables. *P* value < 0.05 is considered as statistically significant difference.

## Results

### Patient characteristics

The demographic characteristics of the study population are presented in Table 1. A total of 336 patients were included, more than half of whom were in Omicron dominant group (58%). Two groups infected with Delta and Omicron variants were similar in age, with a median age of 31. There were 23 cases among 336 participants older than 60 years old. 11.6% of patients had underlying medical conditions. More male and obese patients were reported in Delta prevalent group than in Omicron prevalent group (*p* values of 0.01 and 0.002, respectively). The proportion of Omicron cases that were vaccinated was significantly higher than that of Delta cases (*p* < 0.0001). Except for one patient having one time of vaccination, the other 194 patients infected with Omicron variant were vaccinated with at least two doses, while only 26 Delta cases (18.44%) completed the whole course of vaccination. Among patients presenting with at least one comorbidity (obesity, hypertension, diabetes, COPD, asthma, liver diseases, or cancer), obesity and hypertension were the most frequently reported comorbidities (10.4% and 6.5% of all patients, respectively).

**Table 1:** Patient characteristics

	**Total** *n* = 336	**Delta dominance** *n* = 141 (42%)	**Omicron dominance** *n* = 195 (58%)	***p*** **-value**
***Demographic***				
Age (years), median (range)	31 (26–38)	31 (26–40)	31 (27–36)	0.9
Age ≥ 60	23 (6.85)	11 (7.80)	12 (6.15)	0.7
Male	151 (44.94)	75 (53.19)	76 (38.97)	**0.01**
BMI (kg/m^2^), mean ± SD	21.72 ± 2.70	22.33 ± 2.98	21.28 ± 2.38	**0.005**
Residence in HCMC	307 (91.37)	137 (97.16)	170 (87.18)	**0.003**
***Comorbidity***				
Obesity	35 (10.41)	23 (16.31)	12 (6.15)	**0.002**
Hypertension	22 (6.55)	3 (2.13)	3 (1.54)	0.3
Diabetes mellitus	7 (2.08)	2 (1.42)	5 (2.56)	0.7
COPD	3 (0.89)	3 (2.13)	0	0.1
Asthma	6 (1.79)	3 (2.13)	3 (1.54)	1.00
Liver diseases	3 (0.89)	2 (1.42)	1 (0.51)	0.7
Cancer	1 (0.3)	0	1 (0.51)	1.0
***Number of vaccine doses***				
0	12 (3.57)	12 (8.51)	0 (0)	** < 0.0001**
1	104 (30.95)	103 (73.05)	1 (0.51)	
2	54 (16.07)	26 (18.44)	28 (14.36)	
3	166 (49.40)	0 (0)	166 (85.13)	

Data are presented as sample size (percentage), unless otherwise specified

*BMI* body mass index, *COPD* chronic obstructive pulmonary disease, *HCMC* Ho Chi Minh City

The majority of the participants lived in HCMC (91.4%). During Omicron dominant group, the proportion of patients from other provinces increased 4.5 times compared with Delta group (2.84% and 12.82%, respectively). Similarly, within HCMC, patients in remote areas like districts 7, 10, Binh Thanh and Thu Duc joined telemedicine care at high rates. (Chart S1 and Chart S2).

### Clinical manifestations

Table 2 shows the symptoms that patients experienced by variants. The most common manifestations were fever (64.29%) and those frequently seen in upper respiratory tract infection, including cough (63.39%), sore throat (58.04%) and runny nose (35.12%). The statistically significant differences between Delta dominant group and Omicron dominant group were seen in the prevalence of various symptoms, including runny nose, sore throat, loss of smell or taste, and decreased appetite. The three symptoms that were less common among individuals infected with Omicron variant than people infected with Delta variant are runny nose (*p* = 0.03), loss of smell and taste (*p* < 0.0001) and decreased appetite (*p* = 0.001). Sore throat was significantly more likely to be presented during Omicron prevalence than during Delta prevalence (*p* = 0.03). However, runny nose and sore throat lost their significance after Bonferroni adjustment. Of note, patients recruited during Delta dominant period reported a significantly longer duration of treatment than those in Omicron dominance (median treatment duration of 10 days versus 7 days, *p* < 0.0001).

**Table 2:** Clinical characteristics

	**Total** *n* = 336	**Delta dominance** *n* = 141	**Omicron dominance** *n* = 195	***p*** **-value**	***p*** -value*
SpO_2_ < 95%	48 (14.29)	25 (17.73)	23 (11.79)	0.1	1.0
Fever	216 (64.29)	89 (63.12)	127 (65.13)	0.7	1.0
Shortness of breath	28 (8.33)	13 (9.22)	15 (7.69)	0.7	1.0
Rapid breathing	3 (0.89)	2 (1.42)	1 (0.51)	0.7	1.0
Cough	213 (63.39)	82 (58.16)	131 (67.18)	0.1	1.0
Runny nose	118 (35.12)	59 (41.84)	59 (30.26)	**0.03**	0.42
Sore throat	195 (58.04)	72 (51.06)	123 (63.08)	**0.03**	0.42
Loss of smell or taste	110 (32.74)	88 (62.41)	22 (11.28)	** < 0.0001**	** < 0.0001**
Chest pain	7 (2.08)	3 (2.13)	4 (2.05)	1.0	1.0
Reduced exercise capacity	8 (2.38)	3 (2.13)	5 (2.56)	1.0	1.0
Myoclonus	2 (0.60)	1 (0.71)	1 (0.51)	1.0	1.0
Decreased appetite	46 (13.69)	30 (21.28)	16 (8.21)	**0.001**	**0.014**
Vomiting	3 (8.93)	2 (1.42)	1 (0.51)	0.7	1.0
Duration of treatment (days)	8 (7—11)	10 (7—14)	7 (6—10)	** < 0.0001**	** < 0.001**

Data are presented as sample size (percentage), except for the duration of treatment (median-interquartile range)

*SpO*_*2*_ Saturation of peripheral oxygen

^*^Bonferroni-adjusted

### Clinical outcomes

Clinical outcomes are summarized in Table 3. Overall, no deaths were reported. The majority of participants recovered at home, with only 10/336 (2.98%) of patients requiring hospitalization. Individuals in Delta dominant group were more likely to be hospitalized than those in Omicron dominance (10 cases versus 0 case, *p* = 0.0006).

**Table 3:** Patient outcomes

	**Total** *n* = 336	**Delta dominance** *n* = 141	**Omicron dominance** *n* = 195	***P*** ** value**
At-home recovery	326 (97.02)	131 (92.91)	195 (100)	**0.0006**
Hospitalization	10 (2.98)	10 (7.09)	0 (0)	**0.0006**
Death	0 (0)	0 (0)	0 (0)	1.00

Data are presented as sample size (percentage)

## Discussion

Our study timeline spanned two phases of the COVID-19 pandemic that were characterized by the successive dominance of Delta and Omicron variants. During the first half, medical institutions in HCMC were severely overloaded with confirmed cases, especially critically ill patients. As a result, people with milder symptoms and at-risk populations (i.e., the elderly, people with underlying medical conditions) were much less likely to receive in-person care from healthcare professionals. In response to this situation, the Ministry of Vietnam encouraged the nationwide implementation of telemedicine, while HCMC's Department of Health promptly allowed for their use to provide remote care for people that could safely remain at home [9]. As a private medical facility, Jio Health was able to provide telemedicine service timely for the local community. In contrast, the second half of our study was characterized by the replacement of the less deadly Omicron variant. Nevertheless, people could still develop severe illness, require hospitalization and die from this variant, making home-based self-care without medical help highly unsafe and telemedicine a continuously necessary and attractive option.

Overall, we have demonstrated the safety of telemedicine implementation during both Delta- and Omicron-dominant phases of the pandemic among groups of patients with different demographic and clinical characteristics. The rates of hospital admission and death were low which were comparable to other studies [10, 11] (Table 4). Similar to previous reports, participants in the study were mostly younger adults, showing that this population could be more competent and willing to adapt to technology-based healthcare solutions [12]. Older adults, on the other hand, are also expected to benefit from telemedicine with adequate instruction or assistance from family members. Furthermore, most participants did not have any underlying medical conditions, a result also in line with past studies [13]. People during Delta dominance had a higher rate of comorbidities than those with Omicron dominance, which could be explained by a more popular appeal of telemedicine services among at-risk individuals. Interestingly, while Jio Health offices and clinics were located in HCMC, the service received appointment requests from people living in quite distant provinces, showing that the use of telemedicine surged considerably during Omicron dominant period. Even within HCMC, patients from the remote districts had the highest rates of using telemedicine service. Notably, there were strict policies about distancing within Vietnam and within cities like HCMC, especially in Delta dominant period. Our study, therefore, showed one of the advantages of telemedicine is being able to reach distant populations during a contagious pandemic.

**Table 4:** Telemedicine for COVID-19 patients: hospitalization and death rates

	**n**	**Hospitalization**	**Death**
Our study	336	10 (2.98)	0 (0)
Micallef et al. [10]	156	6 (3.8)	1 (0.6)
Rabunal et al. [11]	275	22 (8)	0 (0)

Data are presented as sample size (percentage)

The majority of participants in the study experienced mild symptoms. The presented clinical manifestations vary among patients infected with Delta and Omicron SARS-CoV-2 variants. Our study showed that COVID-19–highly specific symptoms such as loss of taste and smell were more frequently seen among people with Delta dominance, while those with Omicron dominance were likely to develop symptoms associated with upper respiratory tract infection. This is also consistent with previous findings from the UK and China which reported the prevalence of symptoms associated with an Omicron infection differs from those of Delta variant, apparently with less involvement of lower respiratory tract. This could be due to an adjustment in where the virus survives and multiplies from Delta to the newer Omicron variant [14, 15]. We also found that all hospital admissions in the study occurred during Delta-dominance period and people were sick for a significantly longer time, demonstrating the less severe profile of Omicron variant. This supports other findings from South Africa and South Korea, which showed milder severity and lower risk of hospitalization among people infected with Omicron than those infected with Delta [16–18]. Our finding further suggests that COVID-19 might become endemic in the future and that telemedicine will continue to be an alternative way to deliver safe home-based healthcare for patients and prevent unnecessary hospital visits, as evidenced by a minimal rate of hospitalization (7%) in our study.

There are some limitations in our study. First, the identification of the variant results was not based on genomic sequencing, but on epidemiological findings which could not exclude the overlap between the two variants. Second, the retrospective study design restricted data assessment. Third, the exclusion of pregnant women and the low rate of elderly participants due to concurrent local guidelines for home-based care limited the performance of telemedicine [9]. Fourth, people living in rural areas with inadequate access to healthcare were underrepresented. Fifth, risk factors associated with hospitalizations were not examined.

## Conclusion

Overall, our study demonstrated the role of telemedicine throughout the two phases of the COVID-19 pandemic, which were characterized by the deadly Delta variant causing severe disruption in the medical system, followed by the less lethal but highly transmissible Omicron variant. Telemedicine is a safe resource to care for COVID-19 patients at home.

### Supplementary Information

**Additional file 1: Chart S1.** Locations of participants in Vietnam. **Chart S2.** Locations of participants in Ho Chi Minh City.

## Data Availability

The datasets during and/or analyzed during the current study are available from the corresponding author on reasonable request as per the local ethics committee requirements.

## Abbreviations

BMI: Body mass index
COPD: Chronic obstructive pulmonary disease
COVID-19: Coronavirus Disease 2019
HCMC: Ho Chi Minh City
PCR: Polymerase chain reaction
UMP: University of Medicine and Pharmacy
WHO: World Health Organization
