# Supplementary material for: Telemedicine in home-based care for COVID-19 patients
Source: BMC Prim Care. 2023 Nov 29;24:250. doi: 10.1186/s12875-023-02199-y (PMC10685566; doi:10.1186/s12875-023-02199-y)
Supplement: Supplementary file 1 — Additional file 1: Chart S1. Locations of participants in Vietnam. Chart S2. Locations of participants in Ho Chi Minh City. [file 12875_2023_2199_MOESM1_ESM.docx]

**Telemedicine in Home-based Care for COVID-19 Patients**

*Si Van Nguyen^1,2,*^, Huong Nguyen Viet Duong^1,2^, Hieu Bao Nguyen^[[1]](#footnote-1)^, My Ai Thao Doan^1^, Duc Thanh Nguyen^1^, An Tuan Tran^1^, Khoi Kim Hoang^1^, Oanh Hoang Ly^1^, Thanh Xuan Dang^1^, Tung Ho Thanh Tran^1^, Hung Quang Tran^2^, Nam Ba Nguyen^2^, Thuy Thi Thu Nguyen^1,2^, Raghu Rai^2^,*

*An Le Pham^1^*

**Supplementary Data**

**Chart S1.** Locations of participants in Vietnam

*HCMC: Ho Chi Minh city*

**Chart S2.** Locations of participants in Ho Chi Minh city

*D: district, BT: Binh Tan, BTh: Binh Thanh, GV: Go Vap, PN: Phu Nhuan, TB: Tan Binh; TP: Tan Phu, BC: Binh Chanh, NB: Nha Be, TD: Thu Duc.*

1. [↑](#footnote-ref-1)
